# Mapping residual organics and carbonate at grain boundaries and the amorphous interphase in mouse incisor enamel

**DOI:** 10.3389/fphys.2015.00057

**Published:** 2015-03-19

**Authors:** Lyle M. Gordon, Derk Joester

**Affiliations:** Department of Materials Science and Engineering, Northwestern UniversityEvanston, IL, USA

**Keywords:** dental enamel, caries, atom probe tomography, chemical imaging, grain boundaries, interphases

## Abstract

Dental enamel has evolved to resist the most grueling conditions of mechanical stress, fatigue, and wear. Adding insult to injury, it is exposed to the frequently corrosive environment of the oral cavity. While its hierarchical structure is unrivaled in its mechanical resilience, heterogeneity in the distribution of magnesium ions and the presence of Mg-substituted amorphous calcium phosphate (Mg-ACP) as an intergranular phase have recently been shown to increase the susceptibility of mouse enamel to acid attack. Herein we investigate the distribution of two important constituents of enamel, residual organic matter and inorganic carbonate. We find that organics, carbonate, and possibly water show distinct distribution patterns in the mouse enamel crystallites, at simple grain boundaries, and in the amorphous interphase at multiple grain boundaries. This has implications for the resistance to acid corrosion, mechanical properties, and the mechanism by which enamel crystals grow during amelogenesis.

## Introduction

**Figure 1:**
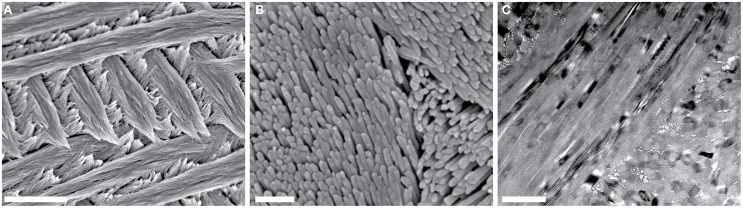
**Overview of mouse incisor enamel structure**. **(A,B)** SEM images of a lactic acid-etched cross-section reveal decussating rods composed of thousands of high-aspect ratio hydroxylapatite nanowires. Scale bars in **(A,B)** correspond to 4 μm and 250 nm, respectively. **(C)** In this bright-field TEM image of a FIB-prepared thin section of the edge of one mouse enamel rod, parallel alignment of nanowires is apparent. Scale bar corresponds to 200 nm.

Enamel, the hardest tissue in vertebrates, is composed of 98 wt% hydroxylapatite (OHAp) along with 1–2 wt% organic molecules and water (Eastoe, 1960). During amelogenesis, ameloblasts first secrete a soft extracellular organic matrix comprised of water (~50 wt%), mineral (~30 wt%), and a number of proteins (~20 wt%), including the amelogenins, ameloblastins, and enamelins, among others (Robinson et al., 1998). As the matrix becomes increasingly mineralized, it is simultaneously processed by proteolytic enzymes, degraded, and largely removed. Nevertheless, it is thought to play an important role in the chemical stabilization of an amorphous mineral precursor, the shaping and mechanical scaffolding of the precursor into a very long and thin ribbon, and the transformation of the precursor into the final crystalline material during enamel maturation (Beniash et al., 2009). Mature enamel is composed of crystallites of OHAp that are highly elongated parallel to the crystallographic *c*-axis and have polygonal cross sections with edge lengths of 20–50 nm in the *a*-*b*-plane. Tens of thousands of crystallites are bundled in rods with a diameter of 3–5 μm. Rods, in turn, are woven together in a complex three-dimensional pattern (Figure 1), with disordered interrod enamel filling the interstices. There are variations of specific aspects of this architecture that depend on the location of the enamel on the crown of a particular tooth, between teeth with different functional morphologies, and between the teeth of different species. Despite these, the similarities remaining are such that rodent teeth, in particular those of rats and mice, are well-established as model systems for amelogenesis and dental caries of human teeth (Bowen, 2013).

Thus, across the species, enamel has a complex composite inorganic-organic architecture with several levels of hierarchy from the nanometer to the millimeter scale (Palmer et al., 2008). Its remarkable hierarchical structure and the constituent organic matter are thought to be responsible for the substantial improvement of enamel mechanical properties, such as toughness, wear resistance, and fatigue life, compared to OHAp. This improvement is integral to enamel function, as pure OHAp would fail catastrophically under the typical forces seen during mastication (Baldassarri et al., 2008). However, enamel has an Achilles heel. It is susceptible to corrosion by acids. Net demineralization of enamel by acids produced in bacterial biofilms in the oral cavity leads to dental caries, the most prevalent infectious disease in humans. Caries severely affects physical and mental health, quality of life, and has major economic consequences (Robinson et al., 2000). It also disproportionately affects children and adults from less affluent backgrounds and in countries with less developed dental hygiene (World Health Organization Media Centre, 2012).

Despite decades of research on enamel, the complex nanoscale structure and chemistry of the tissue is still not fully understood. This has been a major roadblock in the development of accurate models for the mechanism by which carious lesions form, and has effectively prevented the development of early detection schemes, more effective prophylaxis, and minimally invasive therapies. It furthermore imposes limitations on modeling the mechanical properties of enamel and on our understanding of the mechanism by which crystals grow in forming enamel.

For instance, it has long been known that a number of physiologically relevant ions influence crystal growth during enamel maturation and that incorporation of such ions into the mineral phase strongly affects the final structure and physico-chemical properties of enamel. Enamel crystallites consist of OHAp with the prototypical formula Ca_10_(PO_4_)_6_(OH)_2_. However, there is a substantial amount of vacancies, and apatites in general are very tolerant also of substitutional defects. For example, cations such as Na^+^ and Mg^2+^ substitute for Ca^2+^ and anions such as Cl^−^ and F^−^ substitute for OH^−^ (Pan and Fleet, 2002). Carbonate ions (CO^2−^_3_) can replace either hydroxyl or phosphate ions, and are present at such high concentration in biogenic apatites that the latter are frequently referred to as carbonate-hydroxylapatites. The ions with the most substantial effects on the mineral are fluoride, magnesium, sodium, and carbonate ions. Except for fluoride, these substituents increase enamel solubility. However, we have little information regarding the distribution of these ions at length scales below tens of micrometers.

This is because the majority of our understanding is based on bulk compositional analysis, ion- and electron-probe microanalysis, and transmission electron microscopy (TEM). Although these tools have provided valuable information on the structure of enamel, they have a limited ability to probe compositional variations at the length scale of individual crystallites and the interfaces between them. We recently demonstrated that atom probe tomography (APT) is capable of providing this information (Gordon et al., 2015).

**Figure 2:**
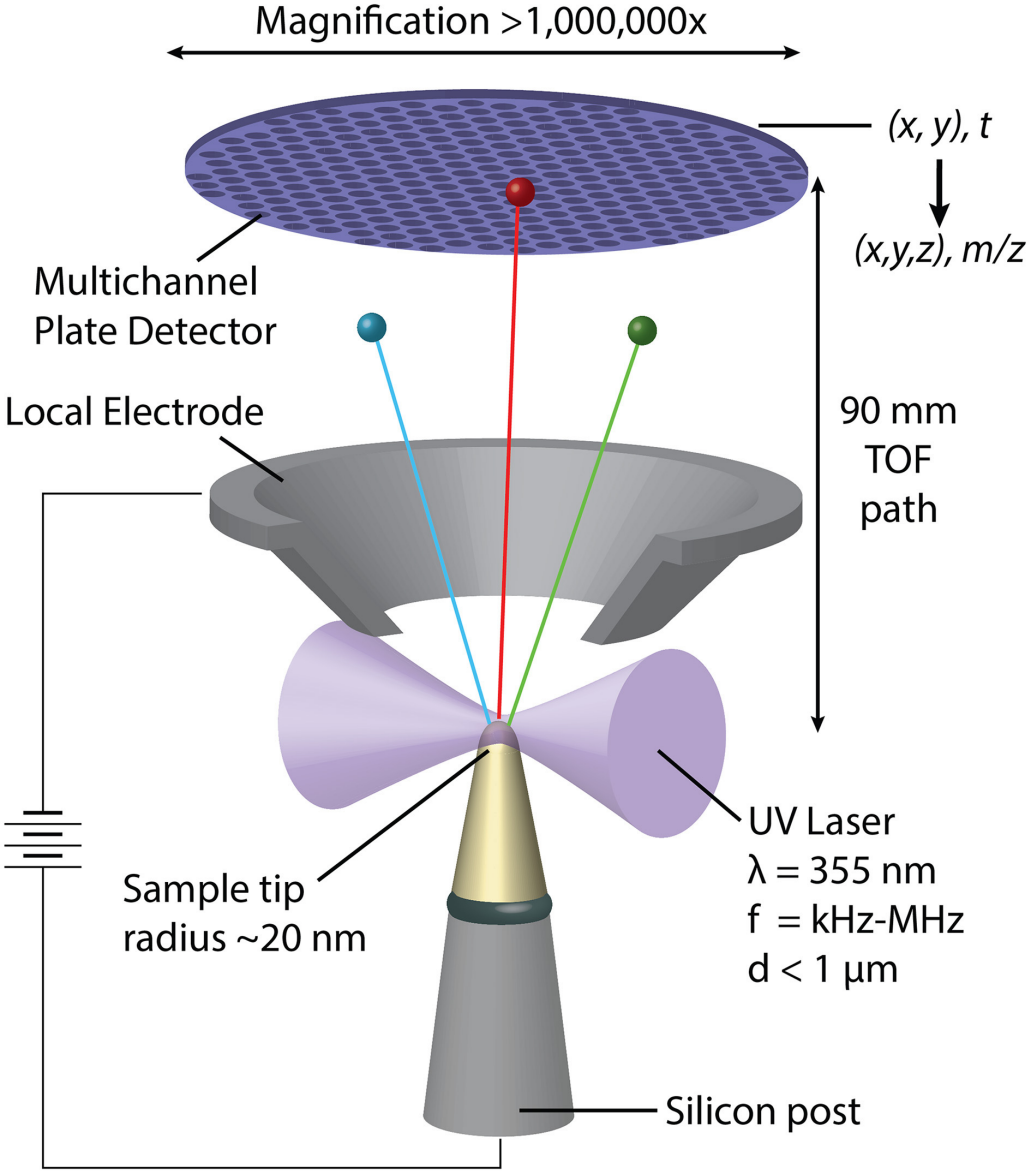
**Schematic representation of a pulsed-laser, local electrode atom probe (LEAP) tomograph**. Following a UV-laser pulse, individual atoms or small clusters at the surface a sharp specimen tip field-evaporate and ionize. Accelerated toward the hollow-cone local electrode, each ion's time of flight is used to determine its mass/charge ratio (m/z) and thus its chemical identity. Where and in what order the ions hit the position sensitive detector depends on their original position in the sample.

In APT, individual atoms or small clusters at the surface of a very sharp, needle shaped specimen are sequentially field-evaporated and -ionized. As the tip is stripped back atom-by-atom and layer-by-layer, each ion is projected onto a position-sensitive detector (Figure 2) (Kelly and Miller, 2007). The mass-to-charge ratio (*m*/*z*) and thus the chemical identity of each ion is determined by time-of-flight (TOF) mass-spectrometry using picosecond laser pulses to trigger evaporation events. The sequence and location of ions impinging on the detector enables reconstruction of the three dimensional structure of the sample. APT analyses volumes on the order of 10^5^–10^6^ nm^3^, typically with sub-nanometer spatial resolution (Kelly and Miller, 2007). Traditionally applied to metals and certain semi-conductors, the development of ultraviolet-laser pulsing greatly increased the scope of the technique to include high resistivity materials and organics (Joester et al., 2012). APT has recently emerged as a technique to characterize hybrid organic/inorganic materials and interfaces, including for instance self-assembled monolayers, invertebrate teeth, and vertebrate bone, dentin, and enamel (Gault et al., 2010; Gordon and Joester, 2011; Gordon et al., 2012; Karlsson et al., 2014).

We used APT to map the distribution of Mg^2+^ in regular, i.e., unpigmented, murine enamel, and found evidence for the presence of Mg-substituted amorphous calcium phosphate (Mg-ACP) as an intergranular phase (Gordon et al., 2015). We further discovered the importance of Mg^2+^ at grain boundaries and in Mg-ACP for the susceptibility of enamel to acid attack. The objectives of the present work were to identify the nanoscale distribution of organic carbon, primarily degradation products of the proteinaceous organic matrix in which the enamel crystallites grow, and inorganic carbon, i.e., carbonate, at enamel grain boundaries and within the amorphous intergranular phase. Both components are thought to strongly impact chemical and mechanical properties of enamel.

## Materials and methods

### Consumables

Epo-Tek 301 epoxy (Epoxy Technology, Billerica, MA); CarbiMet II SiC grinding paper, Microcut SiC grinding paper, Metadi supreme polycrystalline aqueous diamond polishing suspension, Masterprep Alumina suspension, Trident polishing cloth, Chemomet polishing cloth (Buehler, Lake Bluff, IL); Conductive Liquid Silver Paint (Ted Pella, Redding, CA). Super Glue Cyanoacrylate Adhesive (3M, St. Paul, MN).

### Incisors

Incisors were donated by Dr. A. Deymier-Black, Washington University, St Louis, MO.; Dr. C. Newcomb and Dr. S. Sur, Northwestern University. Lower (mandibular) incisors were excised from carcasses of animals euthanized in the context of other studies. After excision, incisors were gently cleaned, rinsed, and dried in air.

### Embedding, grinding, and polishing

Dried incisors were embedded in Epo-Tek 301 epoxy and polymerized overnight at 25°C. Embedded samples were ground using progressively finer grits of Buehler SiC grinding paper (400, 600, 800, and 1200 grit). Ground samples were polished using 3 μm and 1.0 μm polycrystalline aqueous diamond polishing suspensions on a Buehler Trident polishing cloth. After a final polishing step using 0.05 μm Al_2_O_3_ suspension on a Buehler Chemomet polishing cloth, samples were rinsed with water and dried under flowing argon gas.

### Qualitative acid etching

Freshly polished, epoxy-embedded enamel cross-sections were etched for 1 min at 25°C in 250 mM aqueous lactic acid adjusted to pH 4.0 with NaOH.

### Coating

For SEM imaging, samples were secured to an aluminum stub with cyanoacrylate adhesive, coated with ~5 nm of Pt with an Ion Beam Sputter Deposition and Etching System (IBS/e, South Bay Technologies, San Clemente, CA) operating at a base pressure of < 10^−4^ Pa and working pressure of 10^−2^ Pa Ar, with two ion guns operating at 8 kV and 3 mA per gun. The coating was grounded to the stub with conductive liquid silver paint.

### Scanning electron microscopy

SEM was performed with an FEI Helios Nanolab (Hillsboro, OR) operating at 5 keV with 0.1–0.7 nA probe current.

### Transmission electron microscopy sample preparation

Transmission electron microscopy (TEM) lamellae were prepared from a polished mouse incisor cross section following established procedures with a DualBeam scanning electron microscope (SEM) and focused ion beam (FIB) instrument (Helios NanoLab, FEI, Hillsboro, OR) (Giannuzzi and Stevie, 1999). A strap of platinum (FIB-Pt) was deposited over a region of interest using the ion beam (30 kV, 93 pA) to locally decompose an organometallic precursor gas, (trimethyl)methylcyclopentadienyl-platinum [(CH_3_)_3_Pt(CpCH_3_)]. A trench was then milled out (30 kV, 6.5 nA) on either side of a 2 μm wide slice of material. The slice of material was cut free (30 kV, 2.8 nA) from the substrate on three sides leaving only a small connecting bridge. An *in situ* tungsten nanomanipulator probe (Omniprobe) was attached to the free side of the substrate using FIB-Pt (30 kV, 93 pA). The remaining connection to the substrate was milled away (30 kV, 93 pA) and the probe was retracted with the sample. The sample was then welded to a copper TEM half-grid (Omniprobe) using FIB-Pt and the connection to the probe was milled away (30 kV, 93 pA). The lamella was successively thinned to ~100 nm at 30 kV (93 pA) at a 1–2° angle grazing incidence milling condition. The sample was then thinned to ~60–80 nm by low angle milling (~7°) at 5 kV and 2 kV (28 pA); this step also removed the majority of any amorphized/gallium-implanted surface layers.

### Transmission electron microscopy

TEM was performed with a Hitachi H-7700 (Hitachi High-Technologies Science America, Northridge, CA) operating at 120 kV.

### Atom probe tomography sample preparation

Samples for APT were prepared using the dual-beam SEM/FIB instrument (Helios Nanolab, FEI, Hillsboro, Oregon) using established protocols (Miller et al., 2005, 2007; Thompson et al., 2007). A rectangular strap of FIB-Pt was deposited over a region of interest (2 × 25 μm^2^) on polished cross-sections. A wedge of material below the Pt strap was cut out on three sides. The wedge was attached to an *in-situ* nano-manipulator (Omniprobe, Dallas, TX) using FIB-Pt before cutting the final edge free. Segments 1–2 μm wide were cut from the wedge and sequentially affixed to the tops of Si posts in an array (Cameca Scientific Instruments, Madison, WI) with FIB-Pt. Each tip was shaped and sharpened using annular milling patterns of increasingly smaller inner and outer diameters. The majority of the amorphized surface region and implanted gallium in the tip surface was removed by milling at 2 kV, 0.4 nA.

### Atom probe tomography

Atom probe tomographic analyses were conducted in a Cameca local-electrode atom-probe tomograph (LEAP 4000XSi, Cameca, Madison, WI) using a pulsed laser (λ = 355 nm, 200–250 kHz, 50–150 pJ per pulse). The DC potential on a microtip during APT was controlled to maintain an evaporation rate of 0.0025 or 0.005 ions per laser pulse. The base temperature of the microtip was maintained at 40 K and the ambient vacuum pressure was below 10^−8^ Pa. Peak ranges were defined as the entire visible peak and background subtraction was performed using built in routines in Cameca integrated visualization and analysis software (IVAS).

Three-dimensional reconstruction of APT data was performed using IVAS based on published algorithms, assuming a hemispherical tip shape (Bas et al., 1995; Miller, 2000). Standard reconstruction parameters, field factor (*k*_f_ = 3.3) and image compression factor (ξ = 1.33) were used with an electric field-dependent tip radius (*r*). The average evaporation field (*F*_e_) of the enamel apatite (14 V·nm^−1^) was determined from SEM and/or TEM images of microtips after APT analysis. Average atomic volumes for the reconstruction were calculated based on the hydroxylapatite crystal structure (Hughes et al., 1989).

For compositional analysis of apatite crystallites the cores of the grains were manually isolated with multiple rectangular prism regions of interest to exclude the grain boundaries and multiple grain junctions. Twenty nine APT data sets from 11 mandibular incisors of 6 mice were collected and analyzed.

## Results and discussion

### Spectral analysis and global composition

**Figure 3:**
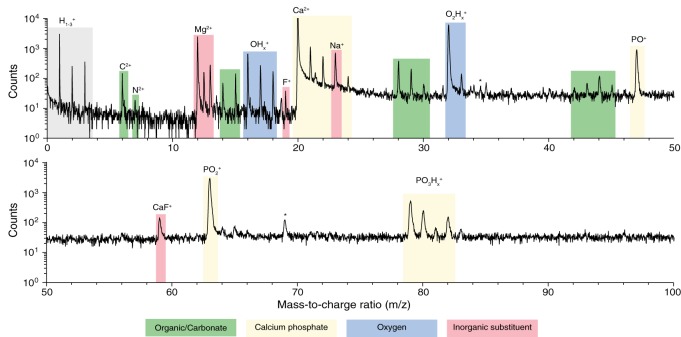
**Atom probe mass spectra**. From APT analysis of isolated organic-rich region at grain edge in mouse inner enamel. Atomic and molecular fragments corresponding to calcium phosphates (mineral), organic molecules, inorganic substituents, and fluoride species, are highlighted. Trace amount of FIB-implanted Ga (^*^) were also detected.

We prepared samples of inner enamel (IE) and outer enamel (OE) from mouse (*Mus musculus)* incisors as described previously (Gordon et al., 2015). Samples for APT were prepared from ground and polished sections by standard focused ion beam (FIB) milling techniques (Thompson et al., 2007). Atom probe spectra (Figure 3) show the typical features of OHAp (Gordon et al., 2012, 2015). Atomic and molecular ions containing Ca, P, and O give rise to a series of peaks of high abundance. Small inorganic cations that are known constituents of enamel, including Mg^2+^ and Na^+^, are present at low abundance. A small amount of fluoride is detected as F^+^ and CaF^+^. This fluoride is likely introduced because of low levels of fluoride in the typical rodent diet.

In addition, spectral features that differentiate enamel from synthetic OHAp were identified. The majority of these correspond to atomic and molecular ions comprised of a combination of one or more of the elements C, H, N, and O (Table 1). Due to the low mass resolving power of the atom probe we cannot unambiguously identify the chemical composition of a number of these ions. For example, at m/z = 28, we would expect to see CO^+^, CH_2_N^+^, and C_2_H^+^_4_. We think that the latter two possibilities are less likely because in H-containing species one typically sees a series of ions corresponding to a parent ion and between zero and a maximum number of hydrogens. However, peaks corresponding to CH_0–2_N^+^ and C_2_H^+^_0–3_ are not found in enamel spectra. While similar arguments can be made in identifying the most likely candidates for many CHNO-containing ions in the spectra, we cannot be certain. As a result, it is not generally possible to determine the chemical identity of the organic molecules present. Indeed, the CHNO-containing ions detected in atom probe spectra of enamel resemble those observed in spectra of bone and dentin (Gordon et al., 2012), where the predominant source is collagen, and chiton teeth, where the organic matrix primarily consists of the polysaccharide chitin (Gordon and Joester, 2011).

**Table 1:** **Detected CHNO-containing ions and relative abundance**.

**Ion**	**m/z**	**Mole fraction (at %)**	**Ion**	**m/z**	**Mole fraction (at %)**
C^2+^	6	0.01	**CO^+^**, C_2_H^+^_4_	28	0.1
N^2+^	7	0.01	**CHO^+^**, C_2_H^+^_5_	29	0.1
C^+^	12	0.1	**CH_2_**O^+^, NO, C_2_H^+^_6_	30	0.07
CH^+^	13	0.06	**CNO^+^**, C_2_H_2_O^+^, C_3_H^+^_6_	42	0.02
**N^+^**, CH^+^_2_, CO^2+^	14	0.03	**CHNO^+^**, C_2_H_3_O^+^, C_3_H^+^_7_	43	0.03
**NH^+^**, CH^+^_3_	15	0.06	**CO^+^_2_**, CH_2_NO^+^, C_3_H^+^_8_	44	0.1
			CO_2_H^+^	45	0.08

*Ions in bold face fonts indicate the identity used for mass spectral decomposition (last column of composition table)*.

An additional complication is that carbonate ions are a known constituent of enamel. Carbonate also gives rise to C^2+^ and CO^+^_x_ (*x* = 0, 1, 2) ions in atom probe spectra (McMurray et al., 2011; Gordon et al., 2012, 2015). However, any nitrogen present most likely originates from organic molecules, for instance proteins that are degraded during enamel maturation. The C/N molar ratio of the known proteins in the enamel organic matrix is in the range from 3.5 to 3.8; that of amelogenin, its most abundant constituent, is 3.76. Assuming that proteolytic degradation of the organic matrix does not change the C/N ratio, we can therefore estimate how much of the total organic mass observed in AP spectra is protein based on the amount of nitrogen observed.

There is only one peak, at m/z = 7, which we can unambiguously identify as N^2+^. We can rule out interference from CH^2+^_2_ because there is no evidence of CH^2+^ (m/z = 6.5) or CH^2+^_3_ ions. While we think it likely that N^+^ and NH^+^ contribute to the peaks at m/z = 14 and 15, we expect at least some contribution from CH^+^_2_ and CH^+^_3_ also. We therefore calculate a lower bound of 5·10^−4^ wt% N and 2·10^−3^ wt% total organic carbon (TOC) using only the count of N^2+^ ions. Using all the potential N-containing peaks (Table 1) provides an estimate of the upper bound (0.04 wt% N and 0.15 wt% TOC in OE, Table 2). Note that even the upper bound for TOC is considerably below the bulk average (~1 wt%) (Eastoe, 1960). This may be due to the higher concentration of organics at the periphery of rods and in interrod enamel (Habelitz et al., 2001).

**Table 2:** **Enamel Composition**.

**Constituent**	**Mass fraction (wt%)**			
	**Outer enamel, whole sample**	**OE crystallites ***N*** = **6****	**Inner enamel, whole sample**	**IE crystallites ***N*** = **7****
Ca	50.46 ± 0.06	47 ± 6	37.76 ± 0.06	34 ± 2
P	15.9 ± 0.2	17 ± 3	21.7 ± 0.2	24 ± 1
O	31.6 ± 0.3	33 ± 3	38.0 ± 0.2	40 ± 1
H	0.231 ± 0.003	0.3 ± 0.03	0.159 ± 0.002	0.2 ± 0.03
Na	0.020 ± 0.001	0.5 ± 0.1	0.590 ± 0.003	0.5 ± 0.1
Mg	0.266 ± 0.003	0.04 ± 0.02	0.332 ± 0.004	0.07 ± 0.04
F	0.189 ± 0.008	0.5 ± 0.3	0.44 ± 0.01	0.5 ± 0.2
Cl	0.128 ± 0.003	0.2 ± 0.06	0.12 ± 0.002	0.2 ± 0.06
N_min_ … N_max_	5·10^−4^ … 0.02	–	8·10^−4^ … 0.04	–
TC = TOC + TIC	0.116 ± 0.004	0.1 ± 0.03	0.151 ± 0.004	0.1 ± 0.05
TOC_min_ … TOC_max_	0.002 … 0.08	–	3·10^−3^ … 0.15	–
TIC_min_ … TIC_max_	0.098 … 0.02	–	0.197 … 0.05	–

*TC, total carbon; TOC, total organic carbon was estimated based on N_max_ and the C/N molar ratio of amelogenin (3.76). TIC, total inorganic carbon was estimated as the difference between TC and TOC. Outer and inner enamel whole sample data are each from one tip reported with error based on counting statistics. The composition of N crystallites is reported as mean ± standard deviation*.

### Spatial distribution of minor enamel constituents

**Figure 4:**
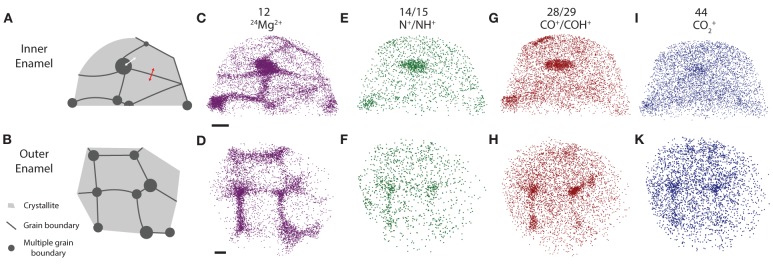
**APT reconstructions. (A,B)** Schematic drawing of grain boundaries between mouse enamel crystallites, **(C,D)** ^24^Mg^2+^, **(E,F)** N^+^, **(G,H)** CO^+^, **(I,K)** CO^+^_2_ ion positions in mouse inner and outer enamel. Scale bars correspond to 10 nm. Composition profiles (Figure 5) are determined by averaging concentrations in the direction normal to a grain boundary, as indicated by the red arrow. Proximity histograms (Figure 5) report average concentrations relative to an isosurface, approximately indicated by white arrow, but integrated over the entire surface. Reconstructions are oriented such that the view direction is parallel to the long axis of the nanowires, i.e., along the [00.1] zone axis.

In three-dimensional reconstructions of mouse enamel (Figure 4), the cross sections of faceted nanowires are readily apparent from the distribution of minor ions such as Mg^2+^ that are present at grain boundaries (Gordon et al., 2015). Grain boundaries are the largely flat interfaces between two adjacent enamel crystallites; grain edges, or more generally, multiple grain boundaries exist where three or more crystallites meet. We previously found that at multiple boundaries, and probably also at simple grain boundaries, an intergranular phase exists that we identified as Mg-substituted amorphous calcium phosphate (Mg-ACP, 0.5–6 wt% Mg^2+^) (Gordon et al., 2015).

Inspection of 3D reconstructions reveals CHNO-containing ions are present at concentrations significantly above the background level at some, but not all multiple grain boundaries and at much lower level throughout the bulk of the grains (Figure 4). There is no evidence of enrichment at simple grain boundaries. It is important to note that while for any given ion identified in atom probe spectra, we can perform a background correction for compositional analysis, it is not possible to decide which of the ions rendered in reconstructions correspond to background. Reconstructions, especially those of low abundance constituents thus appear noisy. Analysis of the nearest neighbor distances (Figure S1) between CHNO-containing ions in OHAp crystallites indicates they are distributed randomly in space (indistinguishable from simulated randomized data). If these ions were fragments of larger organic molecules, we would expect them to cluster. We therefore conclude that there are no organic molecules in the crystallites.

**Figure 5:**
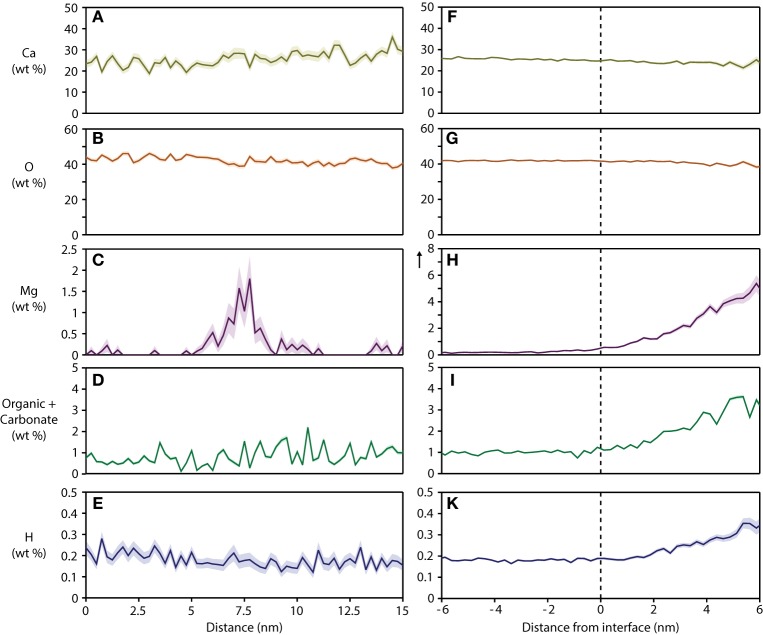
**Concentration profiles and proximity histograms**. Representative concentration profiles across grain boundaries **(A–E)** and proximity histograms of an organic-rich intergranular region **(F–K)** in mouse inner enamel. Note that in proxigrams, distance is plotted relative to the position of the isosurface (0.5 at% Mg), i.e. the interface between the OHAp nanowire and the precipitate. The vertical arrow in plot **(H)** indicates an extended y-axis compared to the corresponding plot **(C)**.

For a quantitative analysis of the distribution of organic matter, we computed one-dimensional concentration profiles (Figures 5A–E) across grain boundaries that were identified on base of the Mg^2+^ distribution. Inspection confirmed the qualitative observation that, unlike Mg^2+^, CHNO-containing ions are not enriched above background at grain boundaries, indicating that neither residual protein, other organics, or carbonate ions are present at higher concentration than in the bulk of the crystallite. As expected, the Ca and O concentration along with the total number of detected ions does not change significantly across the grain boundaries (Figures 5A–B, S2), indicating that there the reconstructions are not affected by trajectory aberrations at the grain boundary (Vurpillot et al., 2000). Given the low absolute concentration of N-containing ions, and the relatively high background, it is likely that the there is in fact no organic material in the bulk or at simple grain boundaries at all.

Based on the absence of organic carbon within the enamel crystallites and at simple grain boundaries, we determined the mass fraction of inorganic carbon (carbonate) within the crystallites. For crystallites in mouse IE and OE, we find 0.1 wt% C (Table 2), corresponding to ~0.5 wt% CO^2−^_3_. For crystalline OHAp this corresponds to 1.2% substitution of CO^2−^_3_ on OH^−^ or PO^3−^_4_ apatite lattice sites. This value is considerably lower than the bulk average of ~3–5 wt% CO^2−^_3_, or 6–10% substitution (Terpstra and Driessens, 1986). It is currently not clear whether this is due to a systematic underestimation of carbonate by APT, or whether carbonate concentrations in enamel are heterogeneous. It is conceivable that carbonate is enriched at the periphery of rods and in the interrod enamel, similar to residual organics.

The composition of the intergranular phase at multiple grain boundaries was quantified by way of an isoconcentration surface (isosurface) that encloses a volume wherein the concentration of a given ion is higher than a threshold (here: 0.5 at% Mg^2+^). Proximity histograms (proxigrams, Figures 5F–K) report the average mole fraction of ions as a function of distance to this isoconcentration surface (Hellman et al., 2000). Such proxigrams indicate enrichment of CHNO-containing ions (1–3 wt%), and therefore carbonate and residual protein, in the intergranular phase. The concentration of carbonate and organic matter at the interface between the crystallites and the intergranular phase is graded over ~3 nm, similar to the concentration of Mg^2+^.

Interestingly, the total concentration of hydrogen also increases from the surface of the crystallite to the interior of the Mg-ACP precipitate. Hydrogen is a ubiquitous contaminant of vacuum chambers made from metals and typically appears in reconstructions as a more or less uniform background. The strong correlation between Mg^2+^, N-, and H-containing ions is atypical and likely corresponds to actual hydrogen present in the sample. Hydrogen can in principle originate from hydroxyl ions, organic matter, and water. An excess of hydroxyl ions would require a mechanism to balance the charge. There is evidence of elevated sodium concentration (~1 wt% at the interphase compared to ~0.5 wt% in the bulk, Figure S3) that could be acting to balance the charge. We therefore think that the increase in hydrogen comes from hydroxyl ions, water or organics, or a combination thereof.

In summary, we conclude that essentially all of the residual organic matter, some carbonate, and possibly some water are present in the Mg-ACP intergranular phase at multiple grain boundaries. Outside the intergranular phase at multiple grain boundaries, the carbonate concentration seems to be homogeneous, and organic matter is most likely absent. This has important consequences for the susceptibility of enamel to acid corrosion that leads to caries lesions, for the mechanical properties of enamel, and provides some information regarding the growth of OHAp crystallites during enamel maturation.

### Implications for acid corrosion of mouse enamel

We have previously shown that grain boundaries in rodent enamel are much more susceptible to acid dissolution than the bulk of the crystallites (Gordon et al., 2015). This leads to highly anisotropic etching and may contribute to the development of subsurface carious lesions. While carbonate and Mg^2+^ are both present at low levels in the bulk of the mouse enamel crystallites, where they are expected to increase solubility (Legfros et al., 1996; Gordon et al., 2015), the high Mg^2+^ content and presence of Mg-ACP at grain boundaries renders these even more soluble. We expect that this effect is exacerbated by the elevated organic and carbonate content of the amorphous interphase. The presence of water would likely allow for more rapid transport of protons into and removal of dissolved ions out of enamel, further weakening its resistance against acid attack. On the other hand, a hydrated amorphous interphase would likely serve as a conduit for rapid diffusion of calcium, phosphate, and fluoride into enamel during re-mineralization from saliva. In fact, the existence of aqueous nanochannels in enamel has been suggested based on transport properties (Featherstone et al., 1979). While the amorphous interphase, at least in atom probe reconstructions, does not appear to be a pore *per se*, it is conceivable that it facilitates diffusive transport processes to a similar degree.

### Implications for enamel mechanical properties

The interfaces between crystallites are of great importance for the mechanical and wear properties of enamel at the nanoscale (He and Swain, 2008; Ang et al., 2010; Arsecularatne and Hoffman, 2012; Yilmaz et al., 2013). A model in which a thin and “soft” organic (mono) layer surrounds each crystallite has been found to fit the experimental data quite well. In contrast to this model, we find organic material only at boundaries of three or more crystallites. However, each crystallite has a “rind” which is high in Mg^2+^, and may indeed be surrounded by a thin intergranular film of Mg-ACP. In this context it is important to note that not all grain boundaries are alike in the sense that the amount and the maximal concentration of Mg^2+^ differ markedly; this may be a function of the specific properties of the grain boundary (angle of misorientation, crystallographic planes). Furthermore, the intergranular phase at multiple grain boundaries is clearly graded and likely has different properties. In ceramics, amorphous intergranular films have been shown to have a dramatic impact on the mechanical properties (Clarke, 1987). Mechanical models of enamel will need to be adjusted to reflect these new insights.

### Implications for crystal growth during enamel maturation

The distribution of Mg^2+^, carbonate ions, organics, and water appears to be markedly different in the bulk of the crystallites, at simple grain boundaries, and in the intergranular phase at multiple grain boundaries. That of Mg^2+^ matches expectations for a species that has low solubility in the crystalline OHAp. During crystal growth from solution, Mg^2+^ would therefore accumulate in the solution ahead of the moving interface. Where the growth fronts of two or more adjacent crystallites meet, Mg^2+^ concentrations in solution would further increase. Some of this Mg^2+^ would be incorporated at grain boundaries. However, if the Mg^2+^ concentration is sufficiently high in the aqueous phase, precipitation of Mg-ACP rather than further growth of OHAp could lead to the formation of the intergranular phase. For carbonate ions, the only difference is that there seems to be no accumulation at simple grain boundaries. This is unusual because it implies that unlike Mg^2+^, it cannot be accommodated there, or that it is sequestered to multiple grain boundaries by a yet unknown mechanism. Organic matter is different in the sense that there seems to be no appreciable incorporation in the crystallites or at simple grain boundaries. This is an interesting finding because proteins of the organic matrix are known to bind to crystalline OHAp *in vitro* and indeed their function is thought to include control over crystal growth (Wallwork et al., 2001). In many other systems, proteins that bind strongly to crystals are occluded during growth (Pokroy et al., 2004; Li et al., 2011). It is possible that the presence of Mg^2+^ in solution modifies their affinity for OHAp, or that proteolytic processing reduces it to the point where they are not incorporated. In any case, the pattern of incorporation of Mg^2+^, organics, and carbonate, and in particular the graded composition of the intergranular phase indicates that the concentrations of these minority constituents in the aqueous phase keeps rising during enamel maturation. This is an important consideration for the design and interpretation of *in vitro* experiments. While we did not find any indication for a direct interaction of residual organics with Mg^2+^ (Gordon et al., 2015), it is possible, even likely that there are specific interactions between organic matter and Mg^2+^, Mg^2+^ and water, or other combinations that play an important role in the formation and the properties of the amorphous intergranular phase.

Note that we interpret our findings here in the context of classical crystal growth from supersaturated solution. Several lines of evidence, however, suggest that initial mineral particles, and possibly even the thin mineral ribbons observed in newly formed, early secretory stage enamel, are comprised of ACP (Landis and Navarro, 1983; Diekwisch et al., 1995; Beniash et al., 2009). ACP therefore is an amorphous precursor phase, similar to those observed in a variety of other systems (Weiner and Addadi, 2011). Typically, amorphous phases are much more accommodating of impurities than crystalline phases. Based on our data, this is true also for ACP, where the amount of Mg^2+^, organics, carbonate, and water is much higher and more variable than in crystalline OHAp. There are in principle three ways for the precursor to transform into the final phase. Dissolution of the more soluble amorphous mineral and re-precipitation of the less soluble crystallites is consistent with the model we discuss above. If the amorphous phase is not a solid, but a dense liquid as observed in protein crystallization (Vekilov, 2004) and suggested for a number of biomineralizing systems (Wallace et al., 2013), the same argument applies. It is much more difficult to reconcile a solid-state phase transformation with the distribution of impurities we observe in mature enamel. This is because impurities trapped in the amorphous precursor at a higher concentration than in the final crystal would have to move away from the crystal growth front; given the low diffusivity of most ions and organic molecules in the solid state at physiological temperatures, this seems kinetically disfavored. We did observe, however, rapid diffusion of fluoride in the amorphous intergranular phase and at grain boundaries at ambient conditions (Gordon et al., 2015), such that it is maybe too early to rule out a solid-state transformation. In addition, more complex mechanisms, such as a mixed mode transformation that involves attachment of amorphous particles at the crystalline interface and concomitant growth and coarsening through the liquid phase remain possible.

## Conclusions

We have shown here that APT allows us to map residual organic matter, carbonate ions, and possibly water to specific nanoscale interfaces and interphases in mouse enamel. We find that all impurities are elevated in the amorphous intergranular phase, Mg-ACP, that is present at multiple grain boundaries, but that the distribution within OHAp crystallites and at simple grain boundaries is specific to the impurity. Based on this distribution we suggest that multiple grain boundaries are particularly sensitive to acid corrosion and are thus expected to play an important role in the development of carious lesions. We furthermore find that individual crystallites are not surrounded by a monolayer of organic matter. Instead, we propose that Mg^2+^ segregation to the grain boundaries and/or an intergranular film of Mg-ACP may be responsible for those aspects of the mechanical performance of enamel that were previously ascribed to the presence of organic matter. Finally, the nanoscale distribution of trace ions, organic, and inorganic carbon in fully mineralized mouse enamel is consistent with crystallization from the amorphous precursor by dissolution and re-precipitation; a solid transformation remains a second possibility. Further work is required to improve our ability to distinguish between organic and inorganic carbon, and to account for the considerable heterogeneity of enamel, for instance the boundaries of enamel rods and interrod enamel. We think that given the fundamental similarities between murine and human enamel, it is likely that many of these findings also apply to the latter. This remains to be proven, however.

### Conflict of interest statement

The authors declare that the research was conducted in the absence of any commercial or financial relationships that could be construed as a potential conflict of interest.
