# Supplementary material for: Mapping residual organics and carbonate at grain boundaries and the amorphous interphase in mouse incisor enamel
Source: Front Physiol. 2015 Mar 19;6:57. doi: 10.3389/fphys.2015.00057 (PMC4365691; doi:10.3389/fphys.2015.00057)
Supplement: Supplementary file 1 [file DataSheet1.DOCX]

**Supplemental Information**

**
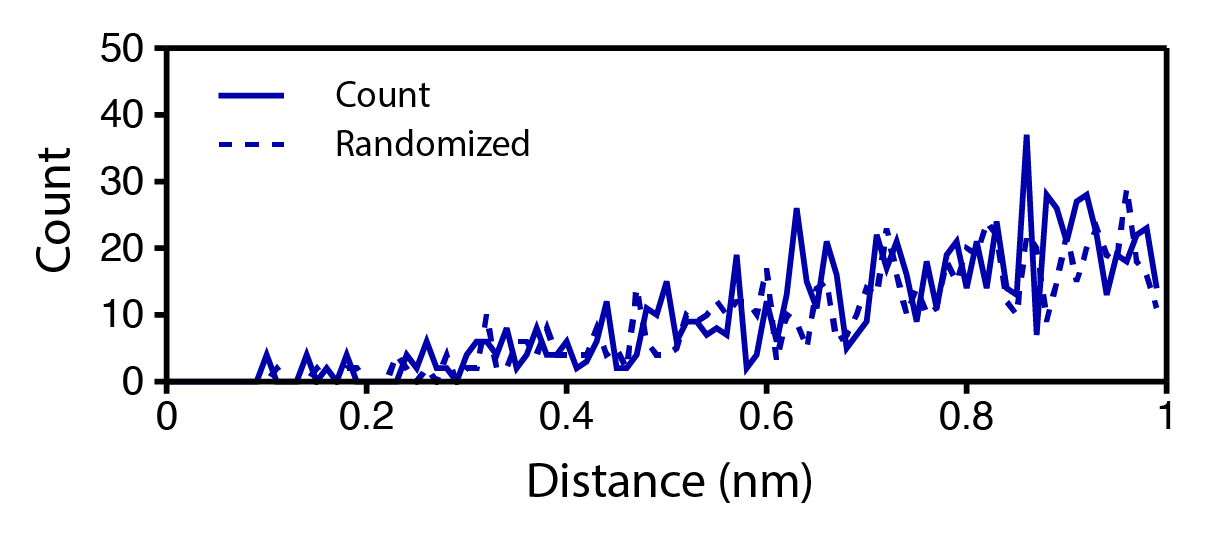
**

**Figure S1:** Nearest neighbor distribution of CHNO-containing ions and simulated randomized data.


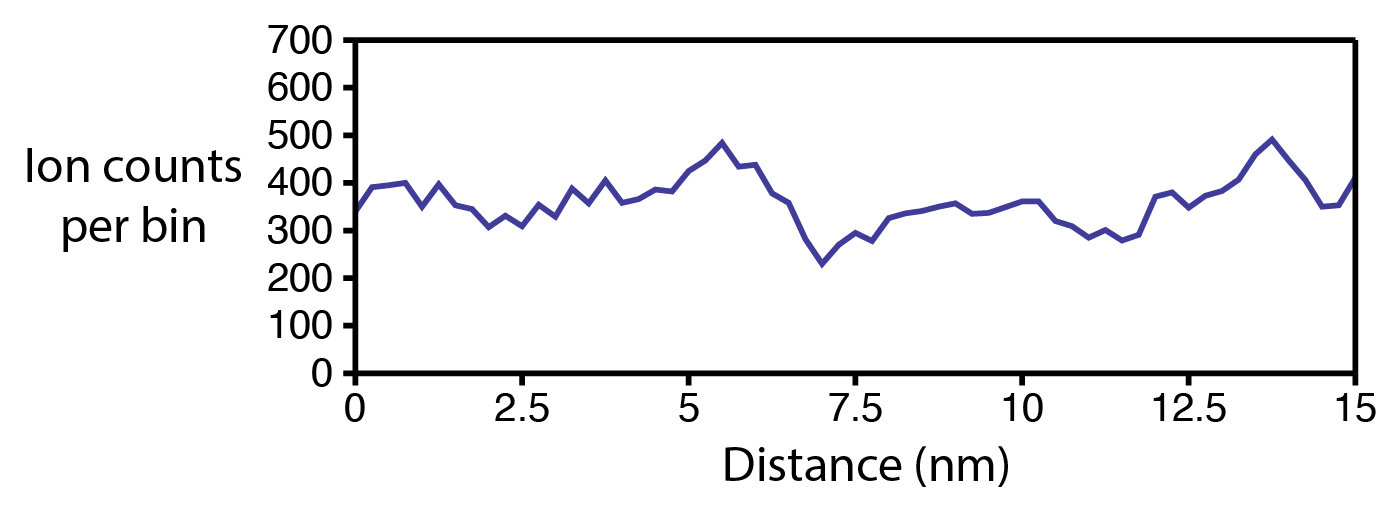


**Figure S2:** Ion counts per bin across grain boundary.

**
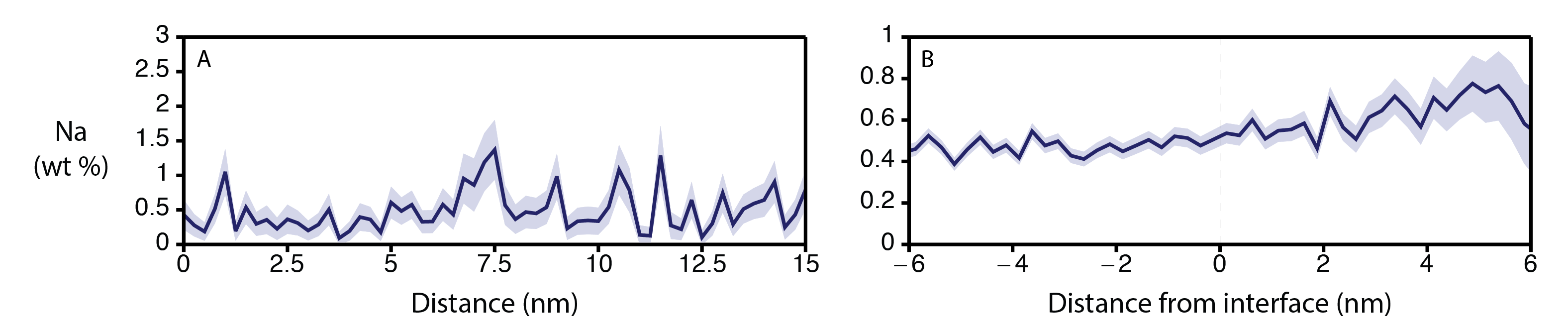
**

**Figure S3:** (A) Sodium 1D concentration profile (wt%) across grain boundary and (B) proximity histogram from multiple grain junction.
